# Global prevalence and associated risk factors of work-related musculoskeletal disorders among steelworkers: a systematic review and meta-analysis

**DOI:** 10.3389/fpubh.2026.1718101

**Published:** 2026-02-05

**Authors:** Yanxiang Lan, Xingxin Zhan, Zhimeng Wang, Dongdong Jiang, Xiaojun Li, Chenchen Peng

**Affiliations:** 1 School of Public Health, Xinyu University, Xinyu, Jiangxi, China; 2 Research Center for Health Promotion and Behavioral Intervention, Xinyu University, Xinyu, Jiangxi, China; 3 School of Public Health, Xinjiang Medical University, Urumqi, China; 4 Fujian Province Key Laboratory of Environment and Health, School of Public Health, Fujian Medical University, Fuzhou, Fujian, China

**Keywords:** global prevalence, meta-analysis, risk factors, steelworkers, work-related musculoskeletal disorders

## Abstract

**Background:**

The prevalence of work-related musculoskeletal disorders (WMSDs) among steelworkers is high due to occupational exposures including high temperatures, vibrating tools, and intense physical loads. Given the varying prevalence estimates and associated risk factors of WMSDs among existing studies and the lack of a meta-analysis dedicated specifically to steelworkers, this meta-analysis aimed to comprehensively evaluate the prevalence of and risk factors associated with WMSDs among steelworkers.

**Methods:**

A systematic literature search of the PubMed, Web of Science, Embase, Scopus, Ovid Medline, Wanfang Data, VIP Database, China National Knowledge Infrastructure, and China Biomedical Literature Service System (SinoMed) databases for published studies reporting the prevalence of and factors associated with WMSDs among steelworkers was performed. Two reviewers independently screened citations, extracted information, and performed quality assessment of the included studies.

**Results:**

Analysis of 35 studies comprising 38,774 participants revealed an annual prevalence of WMSDs of 69.2% (95% CI 56.7%−81.7%) among steelworkers. Subgroup analysis revealed a yearly prevalence rate of WMSDs in Asian nations of 72.1% (95% CI 53.3%−91.0%), which was greater than that for non-Asian countries. The annual prevalence rates reported for 2011–2025 differed significantly from those for 2000–2010 [78.0% (95% CI 67.1%−88.8%) vs. 55.5% (95% CI 40.3%−70.7%), respectively], with lower back injury accounting for the highest annual prevalence [57.2% (95% CI 50.0%−64.5%)], followed by the shoulders and neck [44.7% (95% CI 29.4%−60.0%) and 42.1% (95% CI 27.8%−56.4%), respectively]. At most anatomical sites, except the elbow and hip/leg, the 12-month prevalence was higher than the 7-day prevalence. Risk factors for WMSDs among steelworkers included age ≥30 years, smoking, psychosocial vulnerability, night-shift work, prolonged working hours, use of vibrating tools, lifting heavy loads, low education, high-risk jobs, and poor posture. Scheduled rest breaks were a protective factor.

**Conclusion:**

Steelworkers exhibited a substantial annual prevalence of WMSDs (69.2%), predominantly affecting the lower back, neck, shoulders, and knees. Prioritized implementation of integrated interventions is critical for ergonomic tool optimization, mechanical lifting assistance, targeted health training programs, and systematic high-risk group surveillance to reduce disease burden and safeguard worker wellbeing.

**Systematic review registration:**

[https://www.crd.york.ac.uk/prospero], identifier [CRD420251065458].

## 1 Background

Work-related musculoskeletal disorders (WMSDs) are diseases and injuries involving the neurological, skeletal, and muscular systems caused by occupational activity (1). These disorders include pain, numbness, stiffness, and functional limitations in key anatomical areas (e.g., neck, shoulders, and lower back), which can substantially reduce work productivity and negatively impact quality of life across various occupational groups (2). Workers in the iron and steel industries incur a significant health burden from WMSDs (3) because they are simultaneously exposed to high temperatures, hand–arm vibrations, intense physical labor, and sustained awkward postures (4, 5). Studies have found that steelworkers exhibit substantially higher prevalence rates of WMSDs than those in other manufacturing sectors (6, 7). For example, studies have reported that the annual prevalence rates of WMSDs in the automotive manufacturing industry reaches 53.1% (6) and 50.1% in the electronics industry (7), while among steelworkers, the prevalence is significantly higher, exceeding 90% (8, 9).

Moreover, studies indicate that the global prevalence of WMSDs among steelworkers shows variation in distribution across countries and occupational exposure(s) (10–14). The prevalence of WSMDs among steelworkers exceeds 85% in India and Iran (10, 11), while it is only 25.5% in South Korea (12). Regarding occupational exposure(s), traditional smelting occupations (e.g., foundry, metallurgy) have prevalence rates >70%, with lower rates observed in other occupationally exposed work categories (13, 14). However, systematic reviews summarizing and comparing prevalence rates of WMSDs among steelworkers across different countries remain scarce (15).

Given the high prevalence of WMSDs among steelworkers, numerous researchers have explored various risk factors for WMSDs in this occupational group, including personal characteristics (e.g., smoking, sex), occupational exposure (e.g., heavy lifting, vibration), and psychosocial factors (e.g., prolonged work hours) (16–18). However, current research predominantly focuses on isolated risk factors and devotes little attention to multifactorial interactions. In addition, substantial heterogeneity in geographical settings, methodological approaches, and resulting estimates for similar risk factors, such as vibration, complicate the synthesis of a clear consensus regarding the magnitude and direction of the effect (19). Previous systematic reviews and meta-analyses have examined the prevalence of WMSDs among broader occupational groups, such as metalworkers and manufacturing employees (20, 21). While steelworkers differ from these broader categories, such as metalworkers and manufacturing employees, in their specific job tasks and are subject to a distinct profile of ergonomic risk factors. Therefore, investigating the prevalence of WMSDs specifically within the steel industry is of significant scientific and practical value. However, a comprehensive meta-analysis dedicated to this workforce is still lacking, particularly one that can provide both a global prevalence estimate and an integration of the relevant multifaceted risk factors (e.g., biomechanical, psychosocial, and individual). Therefore, major knowledge gaps persist: (1) a pooled global estimate of WMSDs prevalence among steelworkers, stratified by region and time period (22); (2) a systematic evaluation of the strength and consistency of associations for diverse risk factors unique to steel industry contexts (23); (3) an exploration of the sources of heterogeneity across studies.

To address these gaps, we conducted a systematic review and meta-analysis with the following objectives: (1) to estimate the global prevalence of WMSDs among steelworkers; (2) to identify and quantify key occupational, psychosocial, and individual risk factors for WMSDs in this population; and (3) to explore prevalence variations through subgroup analyses by region, publication year, and job category. Informed by prior studies in related heavy industries, we postulated that the synthesized evidence would likely indicate a high prevalence of WMSDs and would tend to confirm the positive roles of key occupational exposures such as manual material handling and vibration.

## 2 Methods

### 2.1 Study registration

This systematic review and meta-analysis was registered with the International Prospective Register of Systematic Reviews (i.e., “PROSPERO”) database (registration number: CRD420251065458) and adhered to the Preferred Reporting Items for Systematic Reviews and Meta-Analyses (i.e., “PRISMA”) guidelines.

### 2.2 Literature search strategy

A comprehensive, computerized literature search of PubMed, Web of Science, Embase, Scopus, Ovid Medline, Wanfang Data, VIP Database, China National Knowledge Infrastructure (CNKI), and China Biomedical Literature Service System (SinoMed) databases was performed. The search covered the literature published from the respective database inception up to April 19, 2025. Search terms combined Medical Subject Headings (i.e., “MeSH”) and free-text terms, including: “Occupational Musculoskeletal Disease,” “Work-Related Musculoskeletal Disorders,” “WMSDs,” “Musculoskeletal Pain,” “Low Back Pain,” “Steelworkers,” “Foundry Workers,” “Iron Workers,” “Smelting Workers,” “Welders,” and “Metal Workers.” Backward reference tracking of the included studies and relevant reviews was performed to identify additional, potentially eligible studies. The search was not restricted by language. Backward reference tracking of the included studies and relevant reviews was performed to identify additional, potentially eligible studies.

### 2.3 Study inclusion and exclusion criteria

Studies that reported the prevalence of WMSDs were included for the primary objective (prevalence estimation); a subset of these that also reported quantitative risk factor data (e.g., odds ratios) were included for the secondary objective (risk factor analysis). The research question was developed using the Population, Exposure, Outcome (PEO) framework: Population: steelworkers; Exposure: Occupational activities in the steel industry; Outcome: Prevalence and Associated risk of work-related musculoskeletal disorders.

Studies were included based on the following criteria: frontline workers from the steel or metal processing sector (such as those employed in steel making, casting, metallurgy, welding, etc.) constituted the study population; clinical diagnostics or other standardized questionnaires, such as the Nordic Musculoskeletal Questionnaire (NMQ), were used as the evaluation instrument (24); musculoskeletal disorder symptoms (such as pain, numbness, stiffness, or restricted mobility) in nine different body parts (neck, shoulders, elbows, wrists/hands, upper back, lower back, hips/legs, knees, and ankles/feet) over a predetermined time period (usually the previous 7 days or 12 months) were among the criteria for determining WMSDs included in the questionnaire; cross-sectional study design; examined the factors that affect WMSDs among steelworkers and their current status; and outcomes could be extracted directly from the global prevalence of WMSDs in steelworkers or converted into odds ratio (OR) and corresponding 95% confidence interval (CI).

Studies that did not focus on the relevant population or topic, conference abstracts, case reports, reviews, and those with incomplete data, and studies with duplicate data and similar content were excluded.

### 2.4 Literature screening and data extraction

Studies retrieved in the literature search were imported into EndNote X9 (Clarivate, London, United Kingdom) to remove duplicates. Two reviewers independently screened the titles and abstracts, followed by full-text assessment of potentially eligible studies by strictly applying the inclusion and exclusion criteria. Disagreements were resolved by consensus discussion or consultation with a third reviewer. The following information was extracted and cross-checked from the included studies: first author; publication year; country; study population; sample size; prevalence rates; reported risk factors; adjusted ORs with corresponding 95% CIs; and raw data necessary for calculating pooled estimates.

### 2.5 Assessment of study quality

The methodological quality of the included cross-sectional studies was independently evaluated by two reviewers using the 11-item checklist recommended by the US Agency for Healthcare Research and Quality (AHRQ) (25). The evaluation options for each checklist item are “Yes,” “No,” and “Unclear.” A score of 1 is given for “Yes,” and 0 for the others. Studies with a cumulative score ≤ 3 were considered to be low quality, 4–7 medium quality, and 8–11 high quality (26). Inter-rater reliability was assessed with Cohen's kappa, revealing substantial agreement (*K* = 0.72). Discrepancies were resolved by consensus or, when required, arbitration by a third senior reviewer.

### 2.6 Statistical analysis

Statistical analyses were performed using Stata Release 18.0 (StataCorp LLC, College Station, TX, USA). Heterogeneity was assessed using the I^2^ statistic and the χ^2^ test (α = 0.05). A fixed-effects model was applied when *I*^2^ < 50 % and *P* ≥ 0.05; otherwise, a random-effects model was used (27). Subgroup and sensitivity analyses were performed in the presence of heterogeneity to identify the sources and confirm robustness. Publication bias was evaluated using funnel plots and the Egger's test; differences with *P* < 0.05 were considered to be statistically significant. For risk factor synthesis, studies with variable definitions were harmonized before pooling ORs and *I*^2^-based criterion-guided model selection (28). The certainty of evidence for key outcomes was assessed using the GRADE framework for prevalence studies, considering risk of bias, inconsistency, indirectness, imprecision, and publication bias (29).

## 3 Results

### 3.1 Literature search results

**Figure 1:**
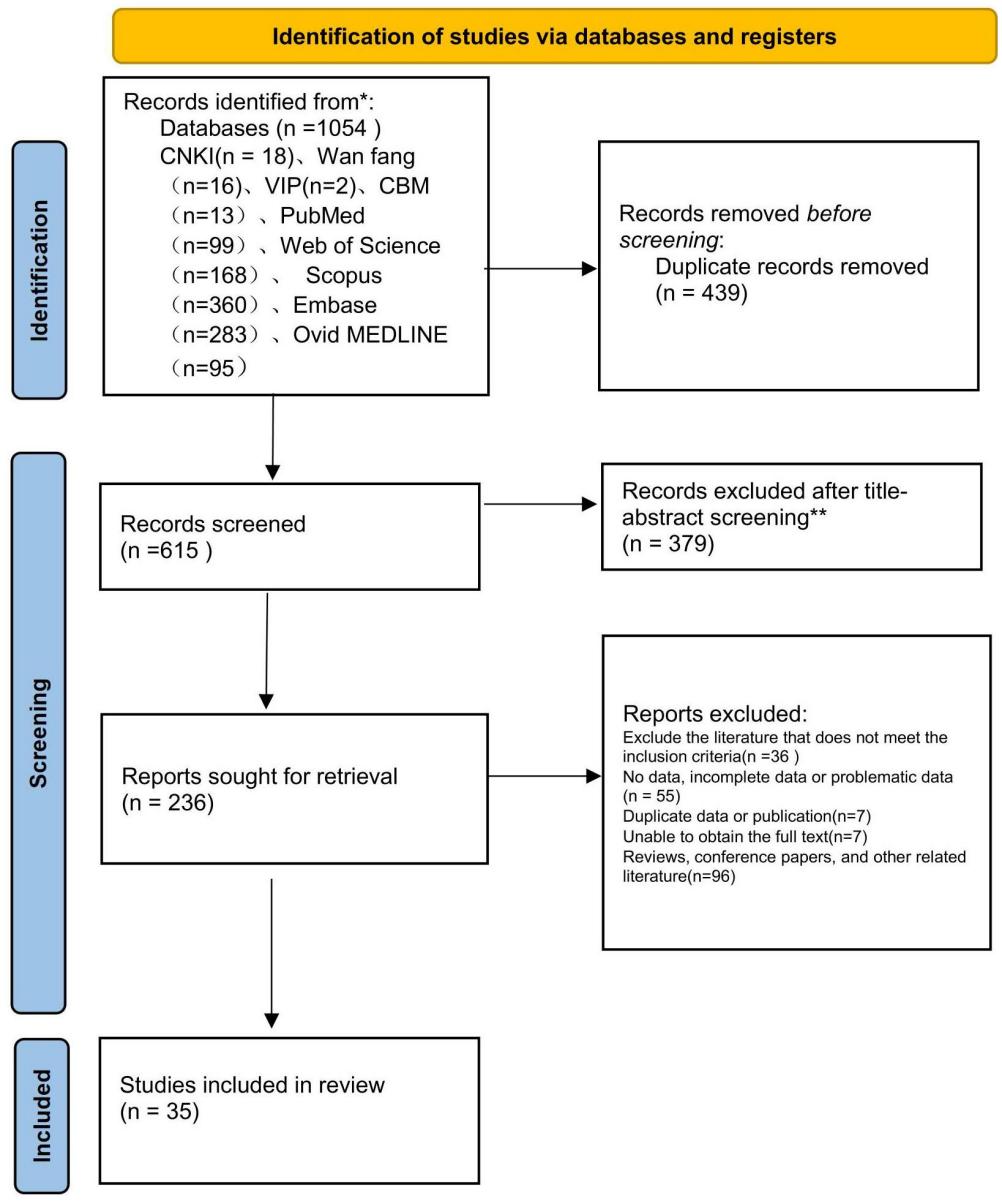
Literature selection process. The single asterisk (*) denotes a footnote that provides details on the databases and/or registries included in the search. The double asterisks (**) refer to a footnote explaining the specific criteria or procedures applied during the title and abstract screening stage for record exclusion.

The initial database search yielded 1,054 studies. After excluding 439 duplicates, 615 records were subjected to title and abstract screening, resulting in the exclusion of an additional 379 studies. The full texts of the remaining 236 studies were assessed for eligibility, of which 201 were excluded for specific reasons. Ultimately, the analysis included 35 studies (8–16, 30–55) comprising 38,774 steelworkers. The literature screening process is illustrated in Figure 1.

### 3.2 Study characteristics and quality evaluation

The fundamental attributes and quality assessment parameters of the included studies, including publication year, country, population source, sample size, prevalence, risk factors, and AHRQ scale scores are summarized in Table 1. According to the AHRQ quality-rating standards, 2 studies fulfilled the high-quality criteria, 27 were medium quality, and 6 were low quality. Based on the GRADE assessment, the evidence indicates a high annual prevalence of WMSDs, supported by moderate-quality evidence. Furthermore, scheduled work breaks are associated with a substantially reduced risk of WMSDs, a finding also supported by moderate-quality evidence. Other estimates, such as those for specific body regions, are less certain due to lower-quality evidence. For detailed outcome data, confidence intervals, and specific downgrading reasons, please refer to the Supplementary Table 3.

**Table 1:** Basic characteristics of included literature.

**Study**	**Publication year**	**Country**	**Study population**	**Sample size**	**The prevalence rate of WMSD**			**Risk factors**	**Literature quality score (AHRQ)**
					**Annual%**	**Body parts**			
						**7 days%**	**12 months%**		
Chellappa et al. (11)	2025	India	Integrated steel workers	40	85.0	-	B:75.0′ K:80.0′ LB:72.5′ N:50.0′ S:60.0′ UB:52.5′ W:55.0	-	2
Alves et al. (30)	2024	Portugal	Metallurgical worker	140	-	LB:42.1′ N:45.7′ S:31.1′ W:32.1	LB:69.3′ N:73.6′ W:72.8	-	6
Yao et al. (31)	2023	China	Welder	972	-	-	UB:42.2	③⑤⑧⑬	8
Li et al. (15)	2023	China	Welder	782	-	-	W:44.1	-	6
Yao et al. (32)	2022	China	Welder	677	-	-	N:54.8	①②③④⑤⑪⑬	2
Elvis et al. (33)	2022	Zimbabwe	Welder	128	-	B:61.0′ E:53.1′ LB:78.1′ N:53.1′ S:66.4′ UB:78.1′ W:61.7	-	④⑤⑥⑩⑪	7
Malakoutikhah et al. (9)	2021	Iran	Integrated steel workers	270	94.8	-	-	-	4
Weyh et al. (34)	2020	Germany	Welder	145	-	-	B:9.0′ E:22.8′ K:46.2′ LB:71.0′ N:60.7′ S:55.2′ UB:35.9	④	5
Hao et al. (16)	2020	China	Integrated steel workers	1082	65.6	A:14.4′ B:11.0′ E:14.4′ K:24.8′ LB:41.3′ N:32.16′ S:27.4′ UB:22.6′ W:18.5	A:21.8′ B:15.0′ E:18.8′ K:33.8′ LB:50.7′ N:39.83′ S:33.8′ UB:27.1′ W:23.7	① → ⑥⑦	6
Shahriyari et al. (35)	2020	Iran	Welder	15	-	-	E:26.7′ K:53.3′ LB:53.3′ N:46.7′ S:46.7′ W:40.0	-	4
Liu et al. (36)	2019	China	Welder	412	-	-	B:43.2′ LB:52.2′ N:48.79′ W:40.8	-	6
Dev et al. (37)	2018	India	Welder	60	-	-	A:45.0′ B:48.3′ E:50.0′ K:41.7′ LB:53.3′ N:51.7′ S:53.3′ UB:38.3′ W:56.7	-	6
Mahesa et al. (38)	2017	India	Welder	987	-	A:50.3′ B:51.2′ E:50.8′ K:46.5′ LB:51.9′ N:55.6′ S:82.2′ UB:49.0′ W:75.1	A:52.8′ B:47.6′ E:76.3′ K:79.1′ LB:49.7′ N:57.71′ S:63.7′ UB:50.5′ W:95.9	⑦⑧⑩	4
Hembecker et al. (39)	2017	Brazil	Metallurgical worker	226	53.5	-	E:15.5′ K:24.8′ N:5.8′ UB:13.3′ W:19.0	⑤	5
Rafeemanesh et al. (40)	2017	Iran	Integrated steel workers	358	-	-	LB:32.4	-	3
Concepcion-Batiz et al. (13)	2016	Brazil	Casting worker	35	74.3	LB:37.1′ W:17.1	LB:68.6′ W:40.0	-	3
Akter et al. (41)	2015	Bangladesh	Integrated steel workers	60	85.0	-	A:45.0′ B:48.3′ K:46.7′ LB:76.7′ N:56.7′ S:58.3′ UB:78.3′ W:63.3	-	5
Sharma et al. (14)	2014	India	Casting workers	516	77.3	-	-	-	6
Kumar et al. (42)	2014	India	Welder	209	-	-	LB:66.5	②④⑧⑪	7
Yesil et al. (43)	2013	Turkey	Integrated steel workers	251	-	-	A:32.7′ LB:63.0′ N:27.5′ UB:12.0	-	3
Ebrahimi et al. (10)	2011	Iran	Welder	160	88.3	-	A:33.1′ B:38.1′ E:73.1′ K:63.1′ N:72.5′ LB:69.4′ S:52.5′ UB:47.5′ W:32.5	⑥	6
Sun et al. (44)	2011	China	Casting worker	1340	-	-	LB:58.9	①⑩⑬	3
Choi et al. (12)	2009	South Korea	Integrated steel workers	1836	25.5	-	E:5.6′ N:7.1′ S:12.1′ W:5.8	③⑫	9
Colucci et al. (45)	2009	Brazil	Integrated steel workers	148	-	-	A:16.2′ B:6.1′ E:4.1′ K:26.3′ LB:35.8′ N:21.6′ S:23.6′ W:17.6	-	5
Picoloto and da Silveira (46)	2008	Brazil	Integrated steel workers	268	75.2	A:14.2′ B:8.6′ E:5.6′ K:10.1′ LB:29.1′ N:18.0′ S:21.6′ UB:17.5′ W:12.3	A:22.4′ B:11.9′ E:9.7′ K:20.1′ LB:45.1′ N:34.3′ S:35.1′ UB:34.3′ W:17.9	-	7
Moussavi-Najarkola and Khavanin (8)	2007	Iran	Integrated steel workers	316	91.8	-	-	-	5
Xu (47)	2007	China	Casting workers	617	-	-	LB:29.2	①④⑨⑩⑫	7
Lei et al. (48)	2005	China	Casting workers	617	-	-	A:3.7′ B:3.9′ E:1.9′ K:2.6′ LB:29.2′ N:6.2′ S:10.5′ W:6.5	①④④⑩⑫	6
van Vuuren et al. (49)	2005	South Africa	Integrated steel workers	366	-	LB:41.3	LB:55.7	⑩⑬	4
Guo et al. (50)	2004	Taiwan, China	Integrated steel workers	18942	37.0	-	-	-	6
Morken et al. (51)	2000	Norway	Metallurgical worker	5654	49.0	-	LB:76.0′ N:68.0′ S:67.0	-	6
Hildebrandt et al. (52)	1996	Netherlands	Integrated steel workers	436	-	-	K:28.0′ LB:53.0	-	6
Masset and Malchaire (53)	1994	Belgium	Integrated steel workers	618	-	LB:25.0	LB:53.0	-	6
Malchaire and Rezk-Kallah (54)	1991	Belgium	Casting workers	33	-	-	N:18.2′ LB72.7′ S:24.2′ W:21.2	-	5
Torner et al. (55)	1991	Sweden	Welder	58	-	-	K:41.0′ LB:81.0′ S:76.0	-	7

A, Ankle/Foots; B, Buttocks/legs; E, Elbow; K, Knee; LB, Lower Back; N, Neck; S, Shoulder; UB, Upper Back; W, Wrist/Hand. ①Gender (Woman); ②Age ≥ 30 years; ③Length of service>5 years; ④Smoking; ⑤ Break time; ⑥ Social psychological weakness(Including occupational stress, social support, etc.);⑦ Night shift duty;⑧ Long working hours;⑨ Use a vibrating tool;⑩ Lifting heavy objects; ⑪ Low educational level; ⑫ High-risk occupation; ⑬ Poor posture. -indicates that no relevant data are available or that quantitative synthesis cannot be performed.

### 3.3 Global prevalence of WMSDs among steelworkers

**Figure 2:**
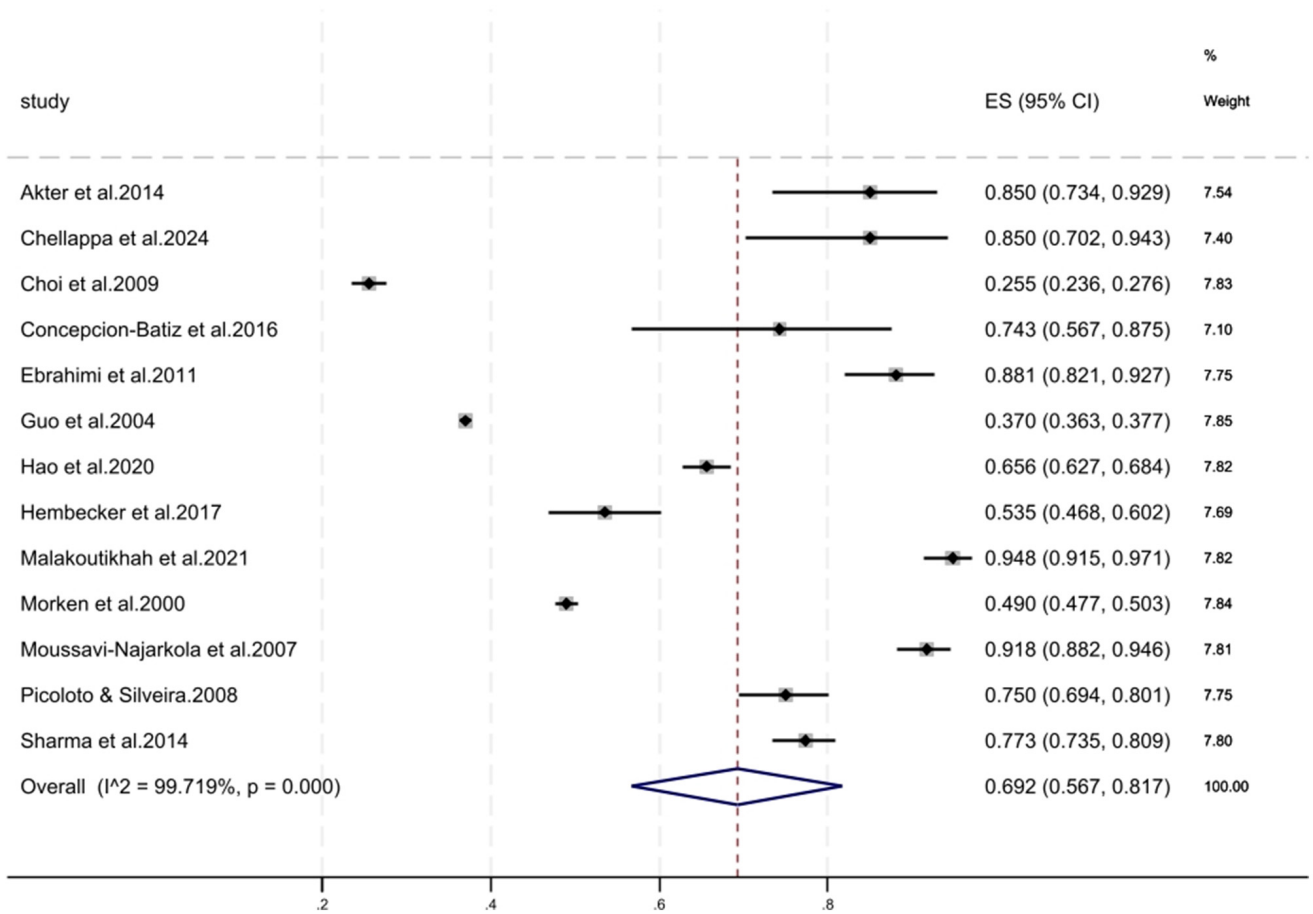
Forest plot of the annual prevalence of WMSDs in steelworkers.

The annual prevalence of WMSDs among steelworkers and their prevalence according to anatomical site were extracted from 35 studies. The annual prevalence rates of WMSDs among steelworkers were compiled using a random-effects model from 13 studies, the combined result was 69.2% (95% CI 56.7%−81.7%) (Figure 2). Prevalence estimates stratified according to anatomical region, time frame (7-day and 12-month periods), and 9 body areas defined by the NMQ are detailed in Table 2, in which data from the 31 studies are summarized. The findings revealed temporal variations in the prevalence of WMSDs across different body regions. The lower back was the most commonly affected site (25 of 31 studies), with 7-day and 12-month prevalences of 43.3% (95% CI 33.3%−53.2%) and 57.2% (95% CI 50.0%−64.5%), respectively. A high 12-month prevalence was also documented for the neck, shoulders, and knees [42.1% (95% CI 27.8%−56.4%), 44.7% (95% CI 29.4%−60.0 %), and 41.7 % (95 % CI 23.8%−59.7%), respectively]. Conversely, the ankle/foot and elbow exhibited the lowest rates, with 12-month prevalences of 30.0% (95% CI 16.4%−43.7%) and 27.5% (95% CI 14.2%−40.7%). When comparing the two time frames, only the elbows, upper back, and buttocks/legs exhibited a slightly lower 12-month prevalence than the 7-day prevalence. For the remaining regions, the 12-month prevalence exceeded the 7-day prevalence. The higher 12-month prevalence across most body regions aligns with the NMQ's design, where the 7-day period captures recent symptoms and the 12-month period picks up chronic or recurring disorders.

**Table 2:** The summary global prevalence of WMSDs in steelworkers stratified by body part and period of time.

**Prevalence: Period of time**	**Neck (%, 95CI, *n*)**	**Shoulder (%, 95CI, *n*)**	**Elbow (%, 95CI, *n*)**	**Wrist/Hand (%, 95CI, *n*)**	**Upper back (%, 95CI, *n*)**	**Lower back (%, 95CI, *n*)**	**Buttocks/ Legs (%, 95CI, *n*)**	**Knee (%, 95CI, *n*)**	**Ankle /Foot (%, 95CI, *n*)**
7-day	40.8 (26.0–55.5) (*n* = 5)	45.8 (16.0–75.6) (*n* = 5)	30.8 (9.3–52.2) (*n* = 4)	36.2 (9.7–62.7) (*n* = 6)	41.6 (20.9–62.4) (*n* = 4)	43.3 (33.3–53.2) (*n* = 8)	32.7 (9.7–55.8) (*n* = 4)	33.2 (16.1–50.3) (*n* = 4)	26.3 (2.4–50.2) (*n* = 3)
12-month	42.1 (27.8–56.4) (*n* = 19)	44.7 (29.4–60.0) (*n* = 15)	27.5 (14.2–40.7) (*n* = 11)	38.3 (16.5–60.2) (*n* = 17)	38.7 (29.1–48.3) (*n* = 11)	57.2 (50.0–64.5) (*n* = 25)	30.6 (19.8–41.5) (*n* = 11)	41.7 (23.8–59.7) (*n* =14)	30.0 (16.4–43.7) (*n* = 9)

### 3.4 Subgroup analyses

Results of subgroup analyses of the annual prevalence of WMSDs among steelworkers are summarized in Table 3. Prevalence was markedly higher in Asian countries [72.1% (95% CI 53.3%−91.0%); *P* < 0.001] than that in non-Asian countries. When studies were stratified according to publication year, prevalence estimates for 2000–2010 [55.5% (95% CI 40.3%−70.7%); *P* < 0.001] differed substantially from those for 2011–2025 [78.0% (95% CI 67.1%−88.8%); *P* < 0.001]. Classification according to job category revealed only minimal differences between integrated steelworkers and frontline workers (e.g., steel making, casting, metallurgy, and welding). The study quality (AHRQ score) indicated an annual prevalence of 71.7% (95% CI 57.0%−86.3%; *P* < 0.001) from medium-quality studies (score 4–7), compared with 61.3% (95% CI 16.8%−105.8%; *P* < 0.001) from low-quality studies and high- quality studies (score ≤ 3 or score 8–11). The subgroup analysis based on sample size revealed that the annual prevalence rate of WMSDs with a sample size of *N* ≥ 500 [50.8% (95% CI 38.6%−63.0%); *P* < 0.001] showed a significant difference from that of *N* < 500 [81.2% (95% CI 72.4%−90.1%); *P* < 0.001].

**Table 3:** The subgroup analysis of annual prevalence of WMSDs among steelworkers.

**Subgroups**		**Number of included studies**	**Heterogeneity test**		**Effect model**	**The prevalence rate of WMSDs/ (95% CI)**
			***I**^2^* **value%**	***P-value***		
Country	Asian countries	9 (8–12, 14, 16, 41, 50)	99.8	< 0.001	Random	72.1 (53.3–91.0)
	Non-Asian countries	4 (13, 39, 46, 51)	99.1	< 0.001	Random	62.4 (47.6–77.2)
Publication year	2000–2010	5 (8, 12, 46, 50, 51)	99.8	< 0.001	Random	55.5 (40.3–70.7)
	2011–2025	8 (9–11, 13, 14, 16, 39, 41)	97.6	< 0.001	Random	78.0 (67.1–88.8)
Job category	Integrated steelworkers	8 (8, 9, 11, 12, 16, 41, 46, 50)	99.8	< 0.001	Random	69.8 (50.1–89.5)
	Front-line steelworkers	5 (10, 13, 14, 39, 51)	99.0	< 0.001	Random	68.3 (50.2–86.5)
Literature quality score (AHRQ)	Low quality or high quality	3 (11–13)	99.8	0.007	Random	61.3 (16.8–105.8)
	medium quality	10 (8–10, 14, 16, 39, 41, 46, 50, 51)	99.6	< 0.001	Random	71.7 (57.0–86.3)
Sample size	*N* ≥ 500	5 (12, 14, 16, 50, 51)	99.7	< 0.001	Random	50.8 (38.6–63.0)
	*N* < 500	8 (8–11, 13, 39, 41, 46)	95.8	< 0.001	Random	81.2 (72.4–90.1)

### 3.5 Results of meta-analysis investigating risk factors for WMSDs among steelworkers

Through data organization, 14 of the 35 studies were included for quantitative analysis, and a total of 13 risk factors were summarized, for which ORs and corresponding 95% CIs were extracted. Significant risk factors (all *P* < 0.05) for the development of WMSDs among steelworkers included age ≥30 years, smoking, psychosocial vulnerability (including social support, occupational stress, etc.), working night shifts, working long hours (>8 h/day or ≥ 40 h weekly), using vibrating tools, lifting heavy objects, having a low level of education (primary or junior high-school), working in high-risk jobs (high physical labor intensity, repetitive motions, exposure to poor posture or heavy lifting, such as turners, welders, porters, etc.), and poor postures (including unnatural positions such as bending, twisting, kneeling, leaning forward, etc.); rest at work was a protective factor (*P* < 0.001); sex and length of service (>5 years) were not significantly correlated (all *P* > 0.05; Table 4).

**Table 4:** Meta-analysis of risk factors for WMSDs among steelworkers.

**Risk factors**	***N***	**Heterogeneity test**		**Effect model**	**OR (95% CI)**	***Z-value***	***P-value***

		***I**^2^* **value(%)**	***P-value***				
Gender (Woman)	5	90.8	< 0.001	Random	1.063 (0.587–1.926)	0.202	0.840
Age ≥ 30 years	2	0.0	0.593	Fixed	1.923 (1.422–2.601)	4.243	< 0.001
Length of service >5 years	3	65	< 0.001	Random	0.802 (0.620–1.037)	−1.683	0.092
Smoking	6	75.4	< 0.001	Random	1.531 (1.170–2.003)	3.108	0.002
Rest at work	4	25.1	0.246	Fixed	0.561 (0.430–0.732)	−4.254	< 0.001
Psychosocial vulnerability	3	90.4	< 0.001	Random	2.489 (1.447–4.281)	3.296	0.001
Night shift duty	2	58.2	0.122	Random	2.971 (1.506–5.860)	3.142	0.002
Long working hours	3	42.9	0.136	Random	1.913 (1.302–2.813)	3.301	0.001
using vibrating tools	2	0	0.694	Fixed	1.932 (1.557–2.397)	5.981	< 0.001
Lifting heavy objects	6	80.2	< 0.001	Random	1.160 (1.034–1.301)	2.532	0.011
Low educational level	3	92.9	< 0.001	Random	4.066 (1.145–14.441)	2.169	0.030
High-risk occupation	3	2.0	0.429	Fixed	2.531 (2.036–3.147)	8.358	< 0.001
Poor posture	4	26.5	0.157	Fixed	1.377 (1.240–1.529)	5.990	< 0.001

For categorical risk factors: The reference group is: Male, Age < 30 years, Length of service ≤ 5 years, Non-smoker, No work breaks, Psychosocial strength, No night shift work, Normal working hours ( ≤ 8h/day or ≤ 40h/week), No use of vibrating tools, Infrequent lifting of heavy loads, Higher education level (tertiary or ≤ 10th grade), Low-risk jobs, Infrequent poor posture.

### 3.6 Sensitivity analysis and publication bias

**Figure 3:**
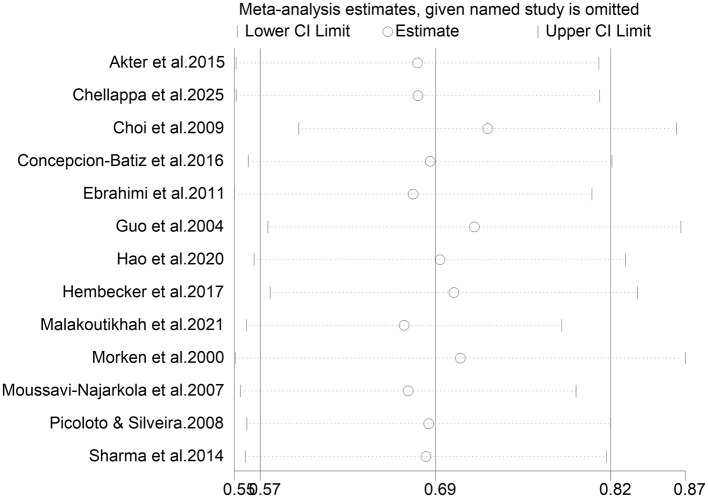
The sensitivity analysis of annual prevalence of WMSDs among steelworkers.

**Figure 4:**
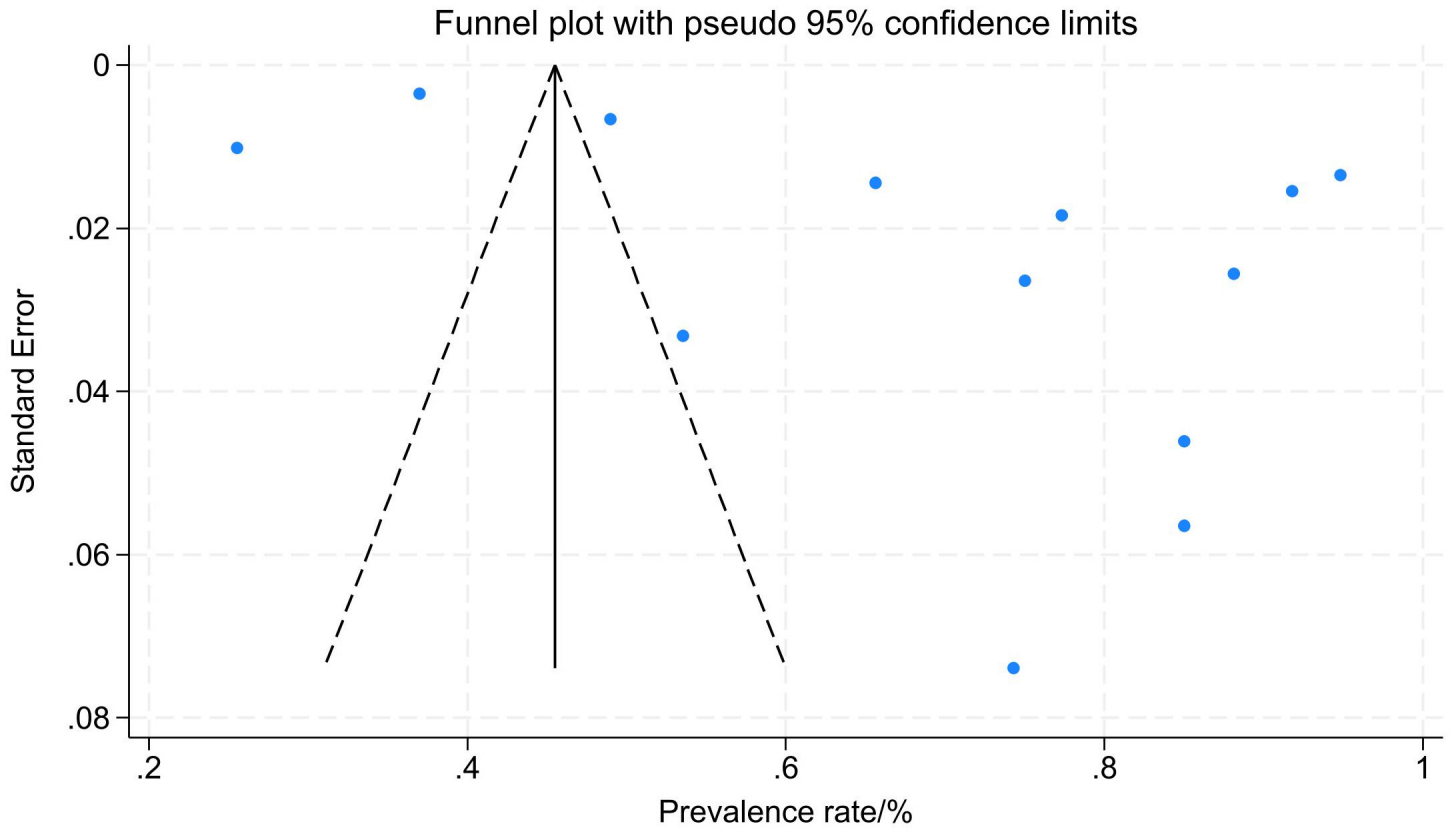
The funnel plot of the annual prevalence of WMSDs in steelworkers.

Due to the high heterogeneity in the combined annual prevalence estimates, a sensitivity analysis was performed. The stepwise removal of individual studies revealed that no single study significantly altered the pooled prevalence or its 95% CI, indicating the stability of the meta-analysis results (Figure 3). The funnel plot exhibited asymmetry and Egger's test was significant (*P* = 0.013), suggesting possible publication bias (Figure 4).

To explore the drivers of heterogeneity and the stability of the primary estimate, studies with specific characteristics were sequentially removed; results are shown in Table 5. In all the examined scenarios, the overall annual prevalence rate remained at a relatively high level, although the specific values varied. Specifically, the estimate decreased to 53.4% (95% CI: 43.7%−63.1%) after excluding studies from high-prevalence regions (Iran, India, and Bangladesh), while it increased to 79.1% (95% CI: 70.4%−87.9%) following the exclusion of studies from low-prevalence regions (South Korea, Norway, Taiwan, and China). Furthermore, by simultaneously excluding regions with high and low disease rates, the heterogeneity was reduced to 88.89%. These analyses indicate that although regional differences do have an impact on the point estimates, the overall conclusion regarding the high incidence rate of occupational musculoskeletal disorders among steel workers remains consistent and robust.

**Table 5:** The annual prevalence of WMSDs among steelworkers under different scenarios.

**Analytical scenario**	**The number of studies**	**The annual prevalence of WMSDs (%)**	**95CI%**	**Effect model**	**Heterogeneity test**	
					***I**^2^* **%**	***p***
Main analysis (all studies)	13	69.2	56.7–81.7	Random	99.72	< 0.001
Excluding studies from Iran	10	62.0	52.4–71.7	Random	99.41	< 0.001
Excluding studies with high prevalence (Iran, India, and Bangladesh)	7	53.4	43.7–63.1	Random	99.39	< 0.001
Excluding studies with low prevalence (South Korea, Norway, Taiwan China)	10	79.1	70.4–87.9	Random	97.45	< 0.001
Excluding studies with low prevalence and high prevalence	4	66.5	57.8–75.1	Random	88.89	< 0.001

## 4 Discussion

The present systematic review and meta-analysis aimed to evaluate the prevalence of WMSDs and associated risk factors among steelworkers worldwide. The synthesis of 35 studies revealed a substantial annual prevalence of WMSDs among steelworkers. This estimate was lower than the 78.0% reported for Western dental professionals (56), but higher than the 53.1% found among workers in China's automobile manufacturing industry, as reported in previous meta-analyses (6). Clearly, this consistently high burden across diverse settings underscores steelmaking as an occupation with uniquely compounded ergonomic hazards, necessitating focused intervention.

Subgroup analysis revealed that the prevalence of WMSDs among steelworkers varied across countries and at different times. The higher annual prevalence observed in Asian countries compared with non-Asian nations likely stems from differences in occupational exposure profiles and work organizations prevalent in many Asian steel industries (57, 58), studies from Iran, India, and Bangladesh have consistently reported exceptionally high prevalence rates (9, 10, 14, 41), which could be linked to a potentially greater reliance on manual labor for tasks, such as material handling and casting, coupled with lower levels of automation compared with settings in Brazil and Europe (13, 39, 46, 51). Furthermore, the implementation and enforcement of comprehensive ergonomic interventions and occupational health policies may be less rigorous or consistent in some rapidly industrializing Asian contexts (59). This geographical disparity is not merely a statistical finding but points to actionable inequities in resource allocation and safety standards. These should tighten rules on ergonomic hazards in the riskiest jobs and advocate for targeted spending on labor-saving technology, such as lifting machines in foundries, and automated carts. Similarly, the temporal increase in prevalence may be explained by a confluence of factors that have evolved over the past 2 decades. First, the steel industry has undergone substantial technological advances and production intensification (60). Consequently, while aiming for efficiency, increased equipment complexity and process refinement in areas such as continuous casting or rolling may cause workers to perform more repetitive tasks or maintain prolonged static or awkward postures (61). Second, the steel-industry workforce in many regions is gradually aging (62, 63). With advancing age, natural declines in musculoskeletal resilience (e.g., reduced muscle mass, bone density, and disc hydration) heighten the susceptibility to injury from sustained occupational exposure(s) (64). Third, compared with earlier studies relying primarily on symptom questionnaires, recent research has increasingly incorporated clinical examinations and imaging techniques to improve the diagnostic rate, potentially identifying subclinical or earlier-stage disorders that may have been previously missed (65). Implementing age management programs and tailoring job assignments for older workers can directly reduce the prevalence of WMSDs, thereby easing the mounting pressure on healthcare services. The subgroup analysis based on sample size revealed a notable difference: studies with smaller samples yielded a substantially higher pooled annual prevalence (81.2%) than those with larger samples. This discrepancy may reflect that smaller studies often focus on specific high-risk workshops or local settings, where selection bias can inflate prevalence estimates (66). In contrast, larger studies typically encompass broader and more representative worker populations, providing a more stable estimate of the disease burden (67). Therefore, the sample size of included studies should be considered when interpreting prevalence estimates. Furthermore, subgroup analysis by study quality (AHRQ score) showed that medium-quality studies presented a pooled prevalence of 71.7%, which was higher than the estimate from the combined group of low- and high-quality studies (61.3%). This pattern may indicate that medium-quality studies strike a balance between methodological rigor and practical feasibility, possibly capturing a more realistic picture of WMSDs in actual workplace settings (6). Some high-quality studies might enroll populations that differ from typical frontline workers due to stricter designs, whereas some low-quality studies may introduce bias through methodological limitations. Future research should aim to improve methodological quality while ensuring sample representativeness and comprehensive reporting.

The global prevalence of WMSDs among steelworkers differs across various body parts, reflecting the biomechanical demands of their work. The lower back accounted for the highest 12-month prevalence of WMSDs, which may be primarily attributed to frequent heavy lifting, forceful bending, and the cumulative strain associated with these tasks, particularly in areas such as material handling and casting (68). Studies show that discs wear out faster when the lower back is loaded past its safe limit, a situation that happens again and again in steel plants during scrap charging or ingot handling, where workers lift heavy weights while bending and twisting the trunk (69). The high prevalences of WMSDs observed in the shoulders, neck, and knees were similar to those reported by Feng et al. (70) in the metal manufacturing industry. This pattern may be associated with sustained static postures and repetitive upper or lower extremity movements that are common in tasks such as welding and assembly (71). In welding, for example, prolonged head tilting and side leaning place chronic strain on the trapezius muscle, contributing to localized pain (72). For the upper limbs, the common prevalence in the elbow (27.5%) and wrist/hand (38.3%) is strongly associated with the use of vibratory tools. The underlying mechanism involves not only muscle microinjury but also nerve compression and local vasoconstriction, which collectively contribute to disorders such as hand-arm vibration syndrome (73). These site-specific injuries highlight the urgent need for targeted ergonomic interventions, such as mechanical lifting aids for the lower back, adjustable workstations, posture rotation for neck/shoulder strain, anti-fatigue matting and knee pads, and anti-vibration tools, coupled with task rotation for the upper extremities.

Work-related psychosocial factors, occupational exposure, and demographic traits are all strongly associated with the incidence of WMSDs (74). Numerous risk variables have been linked to WMSDs among steelworkers. These factors can be broadly classified into three categories: individual, occupational, and psychosocial.

First, older age, smoking, and lower education were associated with increased risk among individual factors. With aging, the cumulative risk of injury rises due to accelerated disc degeneration and reduced muscle suppleness, alongside a general decline in physiological functions such as bone mineral density (64). Smoking significantly impairs bone health by disrupting bone metabolic pathways, altering the equilibrium between bone resorption and bone formation, and causing oxidative stress that harms osteoblasts and slows fracture healing, smoking significantly impairs bone health (75). Supporting this, a study of Chinese males exhibited substantially lower lumbar spine bone mineral density than non-smokers, and smoking increased the levels of biochemical markers of bone and bone turnover in males while also hastening the loss of bone mass (76). Highly educated workers are more likely to obtain occupational protection training and adopt protective work postures, whereas less educated individuals are more likely to work in high-risk positions and have a relatively low awareness of ergonomic protection (74).

Moreover, poor posture, lifting heavy objects, and the use of vibrating instruments are all occupational exposure factors. Carrying heavy loads and using vibrating tools are common tasks performed by steelworkers. These tasks place a great deal of strain on the spine and limbs, particularly excessive bending and twisting, which surpasses the biomechanical tolerance threshold of the spine and accelerates intervertebral disc degeneration and injuries (77). In the context of steel manufacturing, manual material handling tasks, such as charging scrap metal into furnaces or handling cast ingots, require workers to lift substantial loads. When these activities are performed in conjunction with sustained trunk flexion or rotation, they can generate lumbar compressive forces that surpass the biomechanical thresholds associated with accelerated disc metabolic imbalance and annular tears (78). The cumulative effect of repetitive high-load lifting has been implicated in the pathogenesis of lumbar disc degeneration, highlighting the need for ergonomic interventions in heavy industry (79). Microinjuries in the hands and upper extremities are further increased by the use of vibratory equipment, and these cumulative injuries place additional stress on the musculoskeletal system (80). In steel industries, prolonged use of pneumatic grinders, needle scalers, or chipping hammers exposes workers to high-frequency vibration. This can lead to hand-arm vibration syndrome (HAVS), where vascular dysfunction (e.g., episodic digital blanching) and sensorineural damage (numbness, tingling) result from injury to blood vessel linings and loss of the protective coating around peripheral nerves (81). It is frequently necessary to sustain unfavorable posture(s) and position(s) during work, such as the static neck flexion and shoulder abduction common in welding, or the repeated trunk twisting and kneeling in furnace maintenance. Consistent with the results of earlier studies, these positions can cause overstretching and fatigue of muscles and ligaments, increase the strain on joints, and specifically result in overuse or stress concentration in the neck, knees, and lower back, all of which can lead to pain and discomfort (82, 83).

Psychosocial factors contributing to WMSDs in this workforce include high-risk occupations, extended working hours, shift-in-night employment, and psychosocial vulnerability. Psychosocial vulnerability refers to an individual's ability to adjust psychologically to work-related stress and lack of social support. However, steelworkers frequently encounter demanding work pressures, erratic schedules, and intricate work settings, all of which can contribute to elevated psychological stress. According to a study by Ruiyin et al. (84), psychologically stressed workers may have compromised physiological function and resistance, as well as a higher propensity to disregard safety protocols and raise the risk for unintentional injuries. Working night shifts throws off circadian cycles, and bone loss may result from inadequate or poor sleep quality. The physiological processes of the body are comparatively low during night work, and tolerance and muscle and joint flexibility are decreased. The risk for vitamin D deficiency, which lowers bone density and may increase the risk for WMSDs, is also higher among night-shift workers (85). Similar to an Iranian study, long working hours may cause physical exhaustion, particularly in the neck, knees, and lower back (64). Prolonged exposure to high-risk settings further compounds risk, as environmental stressors like heat, noise, and vibration may promote improper posture and movement, increasing physical strain. Conversely, work breaks have been identified as protective factors against WMSDs among steelworkers. Appropriate work breaks can help reduce localized muscle tension caused by both static and exertional loads, which can hinder the evolution of WMSDs (86).

It is noteworthy that there are interconnections between many risk factor categories. Examples of this include occupational exposure, psychosocial variables, and individual factors may all work in concert (87). High physical exertion also implies prolonged labor, which may increase physiological stress. Meanwhile, low social support weakens the psychological recovery ability and together accelerates the development of WMSDs (88). Using vibrating tools in an unnatural position for prolonged periods can increase the burden on specific areas, resulting in poor localized blood circulation and nerve damage, which can lead to overuse and injury of the musculoskeletal system (89). Advancing age may be associated with diminished capacity of the human body to recover from occupational exposure, which could exacerbate preexisting cumulative effects. According to occupational epidemiology research, the “three-dimensional risk model” (biomechanical–psychosocial–person) developed by WMSDs is compatible with the 3 dimensions of individual variables, occupational exposures, and psychosocial factors found in this study (17). Therefore, effective prevention requires integrated and multifaceted intervention programs that simultaneously target biomechanical hazards, optimize work organization, support worker wellbeing, and consider individual vulnerabilities. Based on the aforementioned factors and how they interact, engineering design can improve the workplace in the future by introducing mechanically assisted lifting equipment, ergonomic tools, and reducing the need for physical loads to support workers in maintaining good physical condition and using tools correctly, strengthening individual interventions, such as implementing smoking cessation programs, providing exercise guidance, and occupational health education, and optimizing work arrangements, such as increasing breaks during work, providing more autonomy at work, enhancing social support, and improving job satisfaction.

### 4.1 Practical implications for the steel industry

The reported prevalence and risk profile underscore an urgent, stepwise imperative for evidence-based musculoskeletal prevention in steel plants. Translation of these findings into practice demands a stepped prevention strategy encompassing primary, secondary, and tertiary levels. (1) Primary prevention: intervene at the source by targeting manual materials handling and vibrating tool exposure. Retrofit foundry and storage bays with mechanical assist devices such as ceiling-mounted hoists, roller conveyors, and manipulators, and equip grinding and welding stations with antivibration gloves or reengineered low amplitude tools (90). Complement these with height-adjustable workstations and algorithmic job rotation schedules to interrupt prolonged static loading and repetitive exertion. (2) Secondary prevention: institutionalize enforced, scheduled rest breaks; this inexpensive administrative control attenuates peripheral fatigue and dampens cumulative biomechanical load (16). Deliver concise, literacy-sensitive training modules that demonstrate safe lifting kinematics, optimal working postures, and the dose-response linkage between tobacco use and musculoskeletal vulnerability. (3) Tertiary prevention: embed systematic health surveillance through pre-placement and periodic musculoskeletal screening for workers assigned to high exposure occupations such as welders, casters, and roll operators, and for employees aged 30 years or older (64, 91). Early case detection enables timely clinical intervention and arrests the trajectory toward irreversible functional impairment. Anchored in the hierarchy of controls and aligned with international occupational safety and health frameworks, this integrated strategy furnishes a concrete roadmap for alleviating the disproportionate burden of work-related musculoskeletal disorders among steelworkers.

### 4.2 Alignment with international occupational safety and health frameworks

The findings provide quantitative support for core elements of the ILO Convention C155 and Recommendation R164, which emphasize the responsibility of employers to eliminate ergonomic and other health hazards from the workplace (92, 93). These findings align with the 2021 WHO/ILO technical brief on musculoskeletal disorders, which advocates for a comprehensive approach to addressing these issues (89). Key points of agreement include: (1) systematic assessment of tasks involving heavy lifting, awkward postures, and hand-arm vibration; (2) the use of the hierarchy of controls—prioritizing elimination and engineering measures, with personal protection as a last resort; and (3) routine health surveillance for workers in high-risk jobs to detect early signs of disorders, as outlined in the WHO Global Plan of Action on Workers'Health (94). The pooled estimates can thus guide policymakers and industry stakeholders in applying these international standards and in developing ergonomic guidelines specific to steel production.

## 5 Strengths and limitations

A strength of the present study lies in conducting a comprehensive meta-analysis quantifying the prevalence and synthesizing multifaceted risk factors for WMSDs, specifically among workers in the iron and steel industries. This novel contribution was further enhanced by a rigorous systematic search across 9 major databases, robust quality control via dual-independent screening, validated assessment tools, and stratified subgroup analyses, providing actionable insights. However, this study also had a few limitations. First, the pooled prevalence showed high heterogeneity (*I*^2^ > 90%). Although a random-effects model was used, this spread probably stems from differences in case definitions, recall periods, and the varying ergonomic demands of steelmaking tasks and job titles. The pooled figure is therefore best viewed as a broad summary rather than an exact universal rate. Second, the limited combinability of prevalence measures (point, weekly, lifetime) and insufficient reporting of some variables (e.g., precise exposure levels, body mass index, job-specific experience) hindered more granular analyses. Third, potential publication bias: Egger's test and funnel plot asymmetry suggested possible under-representation of small or non-significant studies, potentially affecting result generalizability. Fourth, the geographical distribution of included studies was uneven, with most data originating from Asia and a notable scarcity of studies from Africa and Latin America. This limits the generalizability of the findings to underrepresented regions. These limitations are systematically reflected in ouGRADE assessment of evidence certainty, which rated the findings as low or very low quality. Consequently, the pooled prevalence estimates presented here should be interpreted as broad, indicative ranges rather than precise global rates. This underscores the need for caution when generalizing the results and highlights the importance of contextual, local data in planning interventions. Future research should prioritize standardized methodologies, detailed reporting of exposures and confounders, longitudinal designs to establish causality, and the inclusion of underrepresented regions to enhance generalizability and comparability.

## 6 Conclusion

In summary, this meta-analysis found an annual prevalence of work-related musculoskeletal disorders among steelworkers of 69.2%, with the lower back, neck, shoulders, and knees most often affected. The risk arose from a combination of individual, biomechanical, and psychosocial factors; scheduled rest breaks were associated with a protective effect. Given the observed variations in prevalence across regions and over time, interventions should be informed by local data. A hierarchical control strategy is proposed: prioritizing engineering controls (e.g., automation, ergonomic tools), followed by administrative measures (e.g., mandated rest breaks, job rotation), and complemented by targeted health surveillance for high-risk groups. Future longitudinal studies are needed to clarify causal relationships, and greater inclusion of participants from underrepresented regions is essential to strengthen the global evidence base.
