# Supplementary material for: Global prevalence and associated risk factors of work-related musculoskeletal disorders among steelworkers: a systematic review and meta-analysis
Source: Front Public Health. 2026 Feb 5;14:1718101. doi: 10.3389/fpubh.2026.1718101 (PMC12916570; doi:10.3389/fpubh.2026.1718101)
Supplement: Supplementary file 1 [file Supplementary_file_1.docx]

Supplementary Material

# Supplementary Figures and Tables

## Supplementary Figures
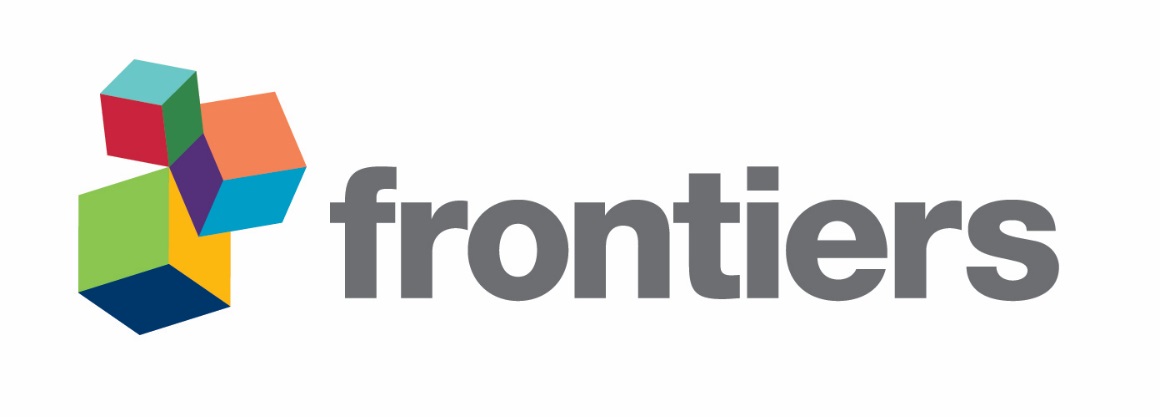


**Supplementary Figure 1.
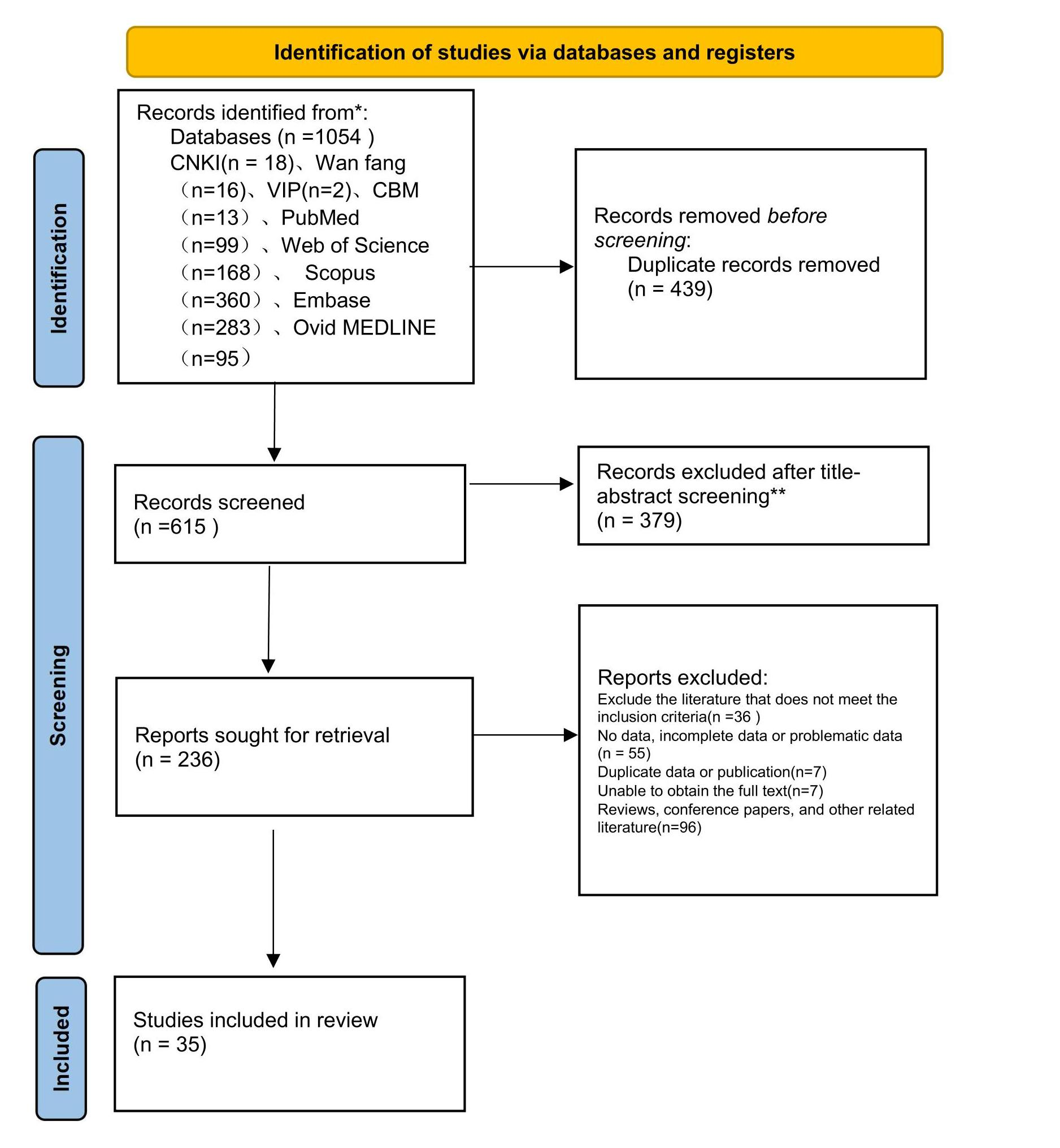
**

Literature selection process

**Supplementary Figure 2.**
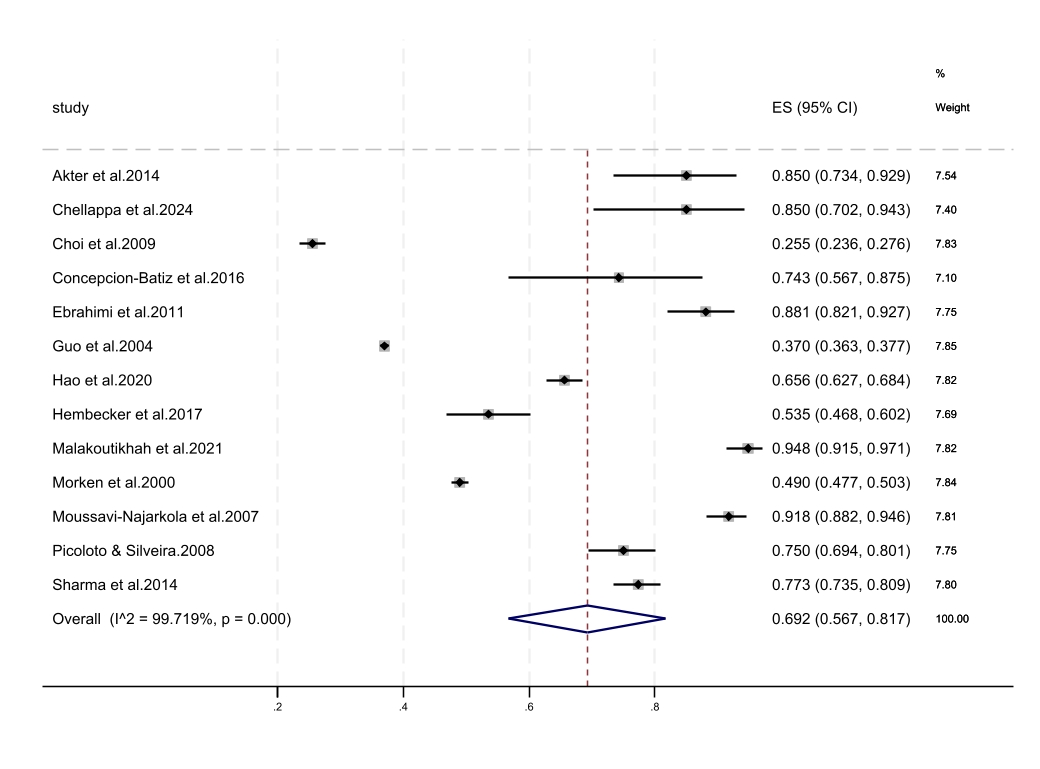


Forest plot of annual prevalence of WMSDs in steelworkers

**Supplementary Figure 3.**


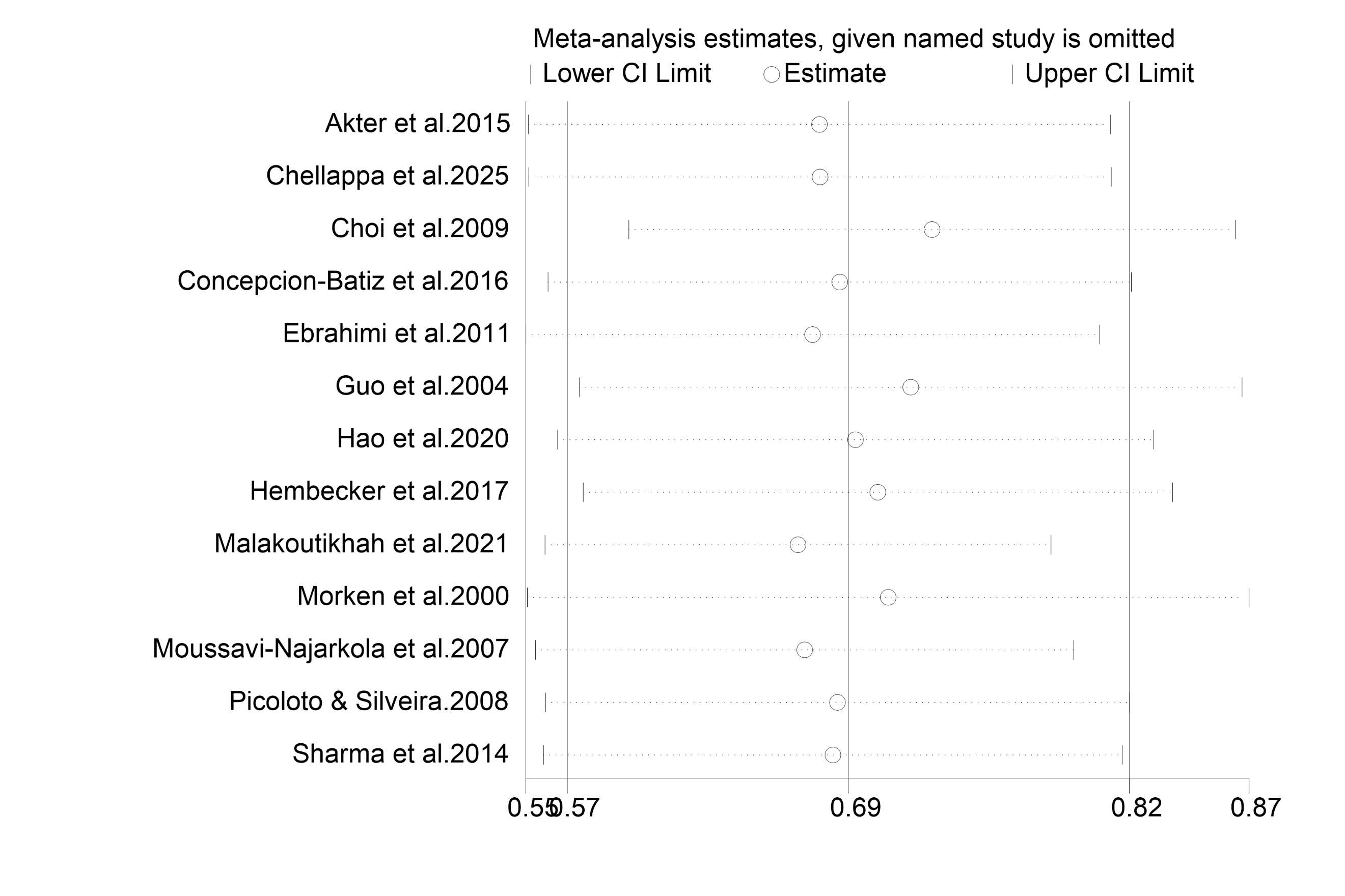


The Sensitivity Analysis of Annual Prevalence of WMSDs among steelworkers

**Supplementary Figure 4.**
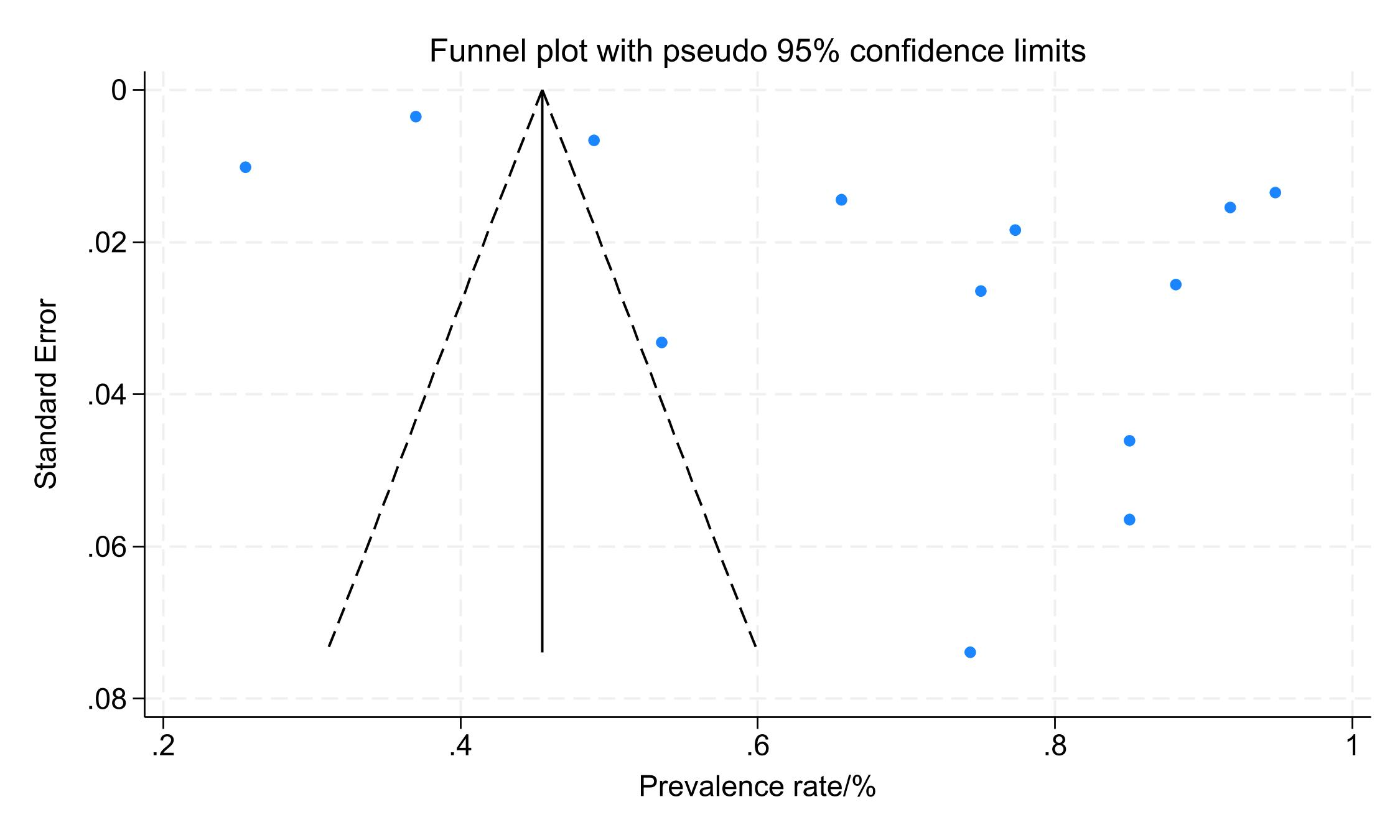


The Funnel plot of the annual prevalence of WMSDs among steelworkers

## Supplementary Tables

**Supplementary Table 1.**

Search formula for work-related musculoskeletal disorders among steelworkers

| Database | Number of literature | Full search term string |
| --- | --- | --- |
| China National Knowledge Infrastructure (CNKI) | 18 | (SU=职业性肌肉骨骼疾患 OR SU=工作相关肌肉骨骼疾病 OR SU=职业性骨骼肌肉疾病 OR SU=腰背痛 OR SU=颈肩痛 OR SU=腕管综合征 OR SU=上肢疾患 OR SU=下背痛综合征 OR SU=肌肉骨骼症状 OR SU=骨骼肌肉劳损 OR SU=职业性腰背疾患)AND(SU=钢铁工人 OR SU=冶金工人 OR SU=冶炼行业 OR SU=轧钢工人 OR SU=重工业 OR SU=炼钢工人 OR SU=铸造工人 OR SU=冶金行业从业者 OR SU=钢铁制造业 OR SU=冶金工业 OR SU=金属加工工人) |
| Wanfang Data | 16 | (主题:(职业性肌肉骨骼疾患) or 主题:(工作相关肌肉骨骼疾病) or 主题:(职业性骨骼肌肉疾病) or 主题:(腰背痛) or 主题:(职业性肌肉骨骼疾患) or 主题:(颈肩痛) or 主题:(腕管综合征) 主题:(上肢疾患) 主题:(下背痛综合征) 主题:(肌肉骨骼症状) 主题:(骨骼肌肉劳损) 主题:(职业性腰背疾患) )and (主题:(钢铁工人) or 主题:(冶金工人) or 主题:(冶炼行业) or 主题:(轧钢工人) or 主题:(重工业) or 主题:(炼钢工人) or 主题:(铸造工人) or 主题:(冶金行业从业者) or 主题:(钢铁制造业) or 主题:(冶金工业) or 主题:(金属加工工人)) |
| VIP Database | 2 | K=(职业性肌肉骨骼疾患 OR 工作相关肌肉骨骼疾病 OR 职业性骨骼肌肉疾病 OR 腰背痛 OR 颈肩痛 OR 腕管综合征 OR 上肢疾患 OR 下背痛综合征 OR 肌肉骨骼症状 OR 骨骼肌肉劳损 OR 职业性腰背疾患) AND K=(钢铁工人 OR 冶金工人 OR 冶炼行业 OR 轧钢工人 OR 重工业 OR 炼钢工人 OR 铸造工人 OR 冶金行业从业者 OR 钢铁制造业 OR 冶金工业 OR 金属加工工人) |
| China Biomedical Literature Service System (SinoMed) | 13 | (("职业性肌肉骨骼疾患"[常用字段] OR "工作相关肌肉骨骼疾病"[常用字段] OR "职业性骨骼肌肉疾病"[常用字段] OR "腰背痛"[常用字段] OR "颈肩痛"[常用字段] OR ("腕管综合征"[常用字段] OR "Carpal Tunnel Syndrome"[常用字段] OR "腕管中央神经病变"[常用字段] OR "腕管压迫性神经病"[常用字段] OR "腕管神经卡压病"[常用字段] OR "腕管综合征"[主题词]) OR "上肢疾患"[常用字段] OR "下背痛综合征"[常用字段] OR "肌肉骨骼症状"[常用字段] OR "骨骼肌肉劳损"[常用字段] OR "职业性腰背疾患"[常用字段]) AND (("钢铁工人"[常用字段] OR "金属工人"[常用字段] OR "Metal Workers"[常用字段] OR "焊工"[常用字段] OR "铁工"[常用字段] OR "金属工人"[主题词]) OR "冶金工人"[常用字段] OR "冶炼行业"[常用字段] OR "轧钢工人"[常用字段] OR ("重工业"[常用字段] OR "冶金学"[常用字段] OR "Metallurgy"[常用字段] OR "重型工业"[常用字段] OR "冶金学"[主题词]) OR "炼钢工人"[常用字段] OR "铸造工人"[常用字段] OR "冶金行业从业者"[常用字段] OR "钢铁制造业"[常用字段] OR "冶金工业"[常用字段] OR "金属加工工人"[常用字段])) |
| PubMed | 99 | ("Occupational Musculoskeletal Diseases" OR "Work-Related Musculoskeletal Disorders" OR "WMSDs" OR "Pain" OR "Musculoskeletal Pain" OR "Low Back Pain" OR "Neck Pain" OR "Shoulder Pain" OR "Carpal Tunnel Syndrome")AND("Steel Industry"OR "Steelworkers" OR "Foundry Workers" OR "Metal Industry" OR "Iron Workers" OR "Smelting Workers" OR "Heavy Industry Workers" OR "Welders" OR "Metal Workers") |
| Web of Science | 168 | ("Occupational Musculoskeletal Diseases" OR "Work-Related Musculoskeletal Disorders" OR "WMSDs" OR "Pain" OR "Musculoskeletal Pain" OR "Low Back Pain" OR "Neck Pain" OR "Shoulder Pain" OR "Carpal Tunnel Syndrome")AND("Steel Industry"OR "Steelworkers" OR "Foundry Workers" OR "Metal Industry" OR "Iron Workers" OR "Smelting Workers" OR "Heavy Industry Workers" OR "Welders" OR "Metal Workers") |
|  |  |  |
| Scopus | 360 | TITLE-ABS-KEY ( "Occupational Musculoskeletal Diseases*" ) OR TITLE-ABS-KEY ( "Work-Related Musculoskeletal Disorders *" ) OR TITLE-ABS-KEY ( "WMSDs *" ) OR TITLE-ABS-KEY ( "Pain *" ) OR TITLE-ABS-KEY ( "Musculoskeletal Pain*" ) OR TITLE-ABS-KEY ( "Low Back Pain *" ) OR TITLE-ABS-KEY ( "Neck Pain*" ) OR TITLE-ABS-KEY ( "Shoulder Pain*" ) OR TITLE-ABS-KEY ( "Carpal Tunnel Syndrom*" ) AND TITLE-ABS-KEY ( "Steel Industry*" ) OR TITLE-ABS-KEY ( "Steelworkers*" ) OR TITLE-ABS-KEY ( "Foundry Workers *" ) OR TITLE-ABS-KEY ( "Metal Industry *" ) OR TITLE-ABS-KEY ( "Iron Workers*" ) OR TITLE-ABS-KEY ( "Smelting Workers*" ) OR TITLE-ABS-KEY ( "Heavy Industry Workers *" ) OR TITLE-ABS-KEY ( "Welders*" ) OR TITLE-ABS-KEY ( "Metal Workers*" ) |
| Embase | 283 | 1#'musculoskeletal disease'/exp OR 'musculoskeletal disease'  2#'occupational musculoskeletal diseases':ab,kw,ti OR 'work-related musculoskeletal disorders':ab,kw,ti OR 'wmsds':ab,kw,ti OR 'pain':ab,kw,ti OR 'musculoskeletal pain':ab,kw,ti OR 'low back pain':ab,kw,ti OR 'neck pain':ab,kw,ti OR 'shoulder pain':ab,kw,ti OR 'carpal tunnel syndrom':ab,kw,ti  3#'metal worker'/exp OR 'metal worker'  4#'steel industry':ab,kw,ti OR 'steelworkers':ab,kw,ti OR 'foundry workers':ab,kw,ti OR 'metal industry':ab,kw,ti OR 'iron workers':ab,kw,ti OR 'smelting workers':ab,kw,ti OR 'heavy industry workers':ab,kw,ti OR 'welders':ab,kw,ti OR 'metal workers':ab,kw,ti  1#OR2#AND3#OR4# |
| Ovid Medline | 95 | (Occupational Musculoskeletal Diseases OR Work-Related Musculoskeletal Disorders OR WMSDs OR Pain OR Musculoskeletal Pain OR Low Back Pain OR Neck Pain OR Shoulder Pain OR Carpal Tunnel Syndrome).ab,ti,kw AND (Steel Industry OR Steelworkers OR Foundry Workers OR Metal Industry OR Iron Workers OR Smelting Workers OR Heavy Industry Workers OR Welders OR Metal Workers).ab,ti,kw |
| Total | 1054 | - |

Filters: The search covered the literature published from respective database inception up to April 19, 2025.

**Supplementary Table 2.**

Agency for Healthcare Research and Quality(AHRQ)

|  | Yes | No | Not clear |
| --- | --- | --- | --- |
| 1. Define the source of information (survey, record review) |  |  |  |
| 2. List inclusion and exclusion criteria for exposed and unexposed subjects (cases and controls) or refer to previous publications |  |  |  |
| 3. Indicate time period used for identifying patients |  |  |  |
| 4. Indicate whether or not subjects were consecutive if not population-based |  |  |  |
| 5. Indicate if evaluators of subjective components of study were masked to other aspects of the status of the participants |  |  |  |
| 6. Describe any assessments undertaken for quality assurance purposes (e.g., test/retest of primary outcome measurements) |  |  |  |
| 7. Explain any patient exclusions from analysis |  |  |  |
| 8.Describe how confounding was assessed and/or controlled. |  |  |  |
| 9. If applicable, explain how missing data were handled in the analysis |  |  |  |
| 10.Summarize patient response rats and completeness of data collection |  |  |  |
| 11.Clarify what follow-up, if any, was expected and the percentage of patients for which incomplete data or follow-up was obtained |  |  |  |

**Supplementary Table 3.**

GRADE Evidence Profile for Key Outcomes

| Outcomes | Anticipated absolute effects(95% Cl) | Number of studies | Quality Of the evidence(GRADE) | Comments |
| --- | --- | --- | --- | --- |
| annual prevalence of WMSDs | 692per1000(567to817) | 13 | ⊕⊕⊕○Moderate | Downgraded by three levels. 1. Risk of bias: Most included studies had moderate methodological quality (AHRQ score 4-7). 2. Inconsistency: Very high heterogeneity (I² > 90%) due to variations in case definitions, recall periods, and job tasks. |
| Lower back prevalence of WMSDs | 572per1000(500to645) | 25 | ⊕⊕○○ LOW | Downgraded by three levels. 1. Risk of bias: As above. 2. Inconsistency: Significant heterogeneity present (I² > 75% in subgroup analyses). 3. Indirectness: As above. The point estimate is relatively precise but context-dependent. |
| Neck prevalence of WMSDs | 421per1000(278to564) | 19 | ⊕○○○Very Low | Downgraded by four levels. 1-3. Risk of bias, Inconsistency, Indirectness: As above. 4. Imprecision: The 95% confidence interval is very wide (28% to 56%), indicating considerable uncertainty around the estimate. |
| Lifting heavy loads | OR 1.16 (1.03 to 1.30) | 6 | ⊕⊕○○ LOW | Downgraded by three levels. 1.Risk of bias:Observational design with potential for confounding. 2. Inconsistency:High statistical heterogeneity (I² = 80.2%) among studies. The effect size is modest. |
| Scheduled work breaks | OR 0.56 (0.43 to 0.73) | 4 | ⊕⊕⊕○Moderate | Downgraded by one level. 1. Risk of bias: Observational design. Not downgraded for inconsistency or imprecision: The effect is consistent (I² = 25.1%) and the confidence interval indicates a precise, clinically important protective effect. |
